# Exploring the prevalence and antibiotic resistance of *Listeria monocytogenes* in diverse food commodities across Sikkim, India

**DOI:** 10.1007/s42770-025-01853-0

**Published:** 2025-12-23

**Authors:** Abhishek Byahut, Madhuchhanda Das, Goutam Chowdhury, Asish Kumar Mukhopadhyay, Rachana Khati, Samaresh Das, Thandavarayan Ramamurthy, Karma G. Dolma

**Affiliations:** 1 Department of Microbiology, Sikkim Manipal Institute of Medical Sciences, Sikkim Manipal University, Tadong, Gangtok, Sikkim 737102 India; 2 Division of Development Research, Indian Council of Medical Research (ICMR) Headquarters, New Delhi, 110029 India; 3 ICMR-National Institute for Research in Bacterial Infections, Kolkata, 700010 India; 4 Center for Development of Advanced Computing (CDAC), Kolkata, 700091 West Bengal India

**Keywords:** Listeria monocytogenes, Foodborne pathogen, Fermented foods, Antimicrobial resistance, MAR index, Sikkim

## Abstract

*Listeria monocytogenes* is a foodborne pathogen of significant public health concern, especially due to its ability to survive in a wide range of foods and cause severe illness in vulnerable populations. This study aimed to investigate the prevalence and antibiotic resistance of *L. monocytogenes* across a variety of food commodities collected from four districts of Sikkim, India, between April 2023 and May 2025. A total of 2,829 samples, including raw meats, cooked foods, fermented products, dairy, street foods, and beverages, were analyzed using standard microbiological methods, followed by confirmation using the VITEK 2 bacterial identification system and PCR assay. The antibiotic susceptibility tests (AST) were done and multiple antibiotic resistance (MAR) index values were calculated. Of these tested samples, 98 (3.46%) were positive for the *L. monocytogenes*. Cooked items and raw meats showed the highest contamination rates, while traditional fermented foods and street food also contributed substantially. District-wise, South Sikkim had the highest number of positive samples. AST revealed notable resistance to penicillin, ampicillin, erythromycin, and clindamycin, with high susceptibility to tetracycline and trimethoprim-sulfamethoxazole. The MAR index values ranged from 0 to 0.81 with 41 isolates showing higher values above 0.2. Our findings highlight the need for improved hygiene practices, regular surveillance, and cautious use of antibiotics. The study underscores the importance of region-specific food safety strategies, particularly in areas where traditional food practices are deeply rooted in daily life.

**Supplementary Information:**

The online version contains supplementary material available at 10.1007/s42770-025-01853-0.

## Introduction

*Listeria monocytogenes* is a foodborne pathogen responsible for listeriosis, which causes fewer infections compared to other foodborne pathogens but results in remarkable mortality rates of 20–30% in high-risk populations worldwide [1, 2]. The disease primarily targets pregnant women together with elderly people and immunocompromised individuals, as well as unborn babies and newborns [3]. *L. monocytogenes* is a Gram-positive, facultatively anaerobic microorganism that exhibits motility within the temperature range of 22–28 °C, but becomes non-motile at temperatures exceeding 30 °C [4]. It can grow in a temperature range from − 0.4 °C to 45 °C, with an optimum growth temperature of 37 °C. It can survive at a low water activity (aW < 0.90) and across a broad pH range of 4.6 to 9.5, in addition to tolerating salt concentrations of up to 20% [5].

Foodborne diseases (FBDs) continue to be a significant burden on public health in India. Annually, there are approximately 100 million cases of FBDs in the nation, which results in a loss of $15 billion in terms of economy [6]. According to the World Health Organization (WHO), South-East Asia, of which India is a part, reports 150 million cases and 175,000 deaths annually, with young children being most affected [7]. India’s primary food safety regulator, the Food Safety and Standards Authority of India (FSSAI), operates under the authority of the Food Safety and Standards Act of 2006 and is responsible for setting and enforcing microbiological standards across food categories [8]. Among these, *L. monocytogenes* is explicitly addressed in the Microbiological Criteria under the Food Products Standards and Food Additives Regulations: zero tolerance of *L. monocytogenes* in 25 g of ready-to-eat foods [9]. Despite this regulatory framework, there are currently no documented outbreaks of listeriosis in India in peer-reviewed or national surveillance reports, which may reflect underreporting or limited surveillance capacity. In India, it is estimated that the transmission of animal-sourced foods is responsible for approximately 21% of total foodborne diseases [6]. To address these challenges, the FSSAI has implemented various programmes such as the ‘Eat Right India’ campaign for ensuring healthier food options, and steps to strengthen the nation’s food testing capacity [10]. Healthy people who contract *L. monocytogenes* infection develop mild gastroenteritis that resolves on its own. Patients with deficient cell-mediated immunity face serious infections including sepsis, meningitis and encephalitis, which result in life-long consequences and even death [11]. Although listeriosis occurs less frequently than many other foodborne zoonotic diseases, its severity and growing resistance make it challenging to eliminate, which raises significant concern [12].

*L. monocytogenes* maintains its survival abilities in refrigerated ready-to-eat and processed foods, which makes it a continuous threat to worldwide food safety [13]. The actual extent of contamination remains unknown because surveillance in resource-limited regions with distinctive dietary patterns remains poorly understood. In India, *L. monocytogenes* has been found in seafood, with a prevalence of 0.55% from Kerala, while milk samples from Maharashtra show excessive contamination with 53.8% of farms, mutton samples from Punjab show 3.2%, and dairy products from Tamil Nadu show 3.5%, indicating high-risk foods with widespread animal-derived and fresh produce contamination across states [14–17]. Foodborne infections have drawn more attention recently because of their significant adverse impacts on public health, especially in north-east India, where the culture, life style and eating habits are different from the rest of the country. Among these infections, *L. monocytogenes* is particularly critical since it can grow in a variety of widely consumed food products. Its presence in local cuisines such as kinema, a traditional fermented soybean product [18], is especially concerning. The rare but serious consequences of human food contamination by *L. monocytogenes* highlight the need to implement monitoring of standard protocols for organisms, especially in different food processing sectors, particularly those involving animal-based products [19]. The need for improved food safety protocols and monitoring has been highlighted by studies on the Hazard Analysis Critical Control Points (HACCP), which found concerning variations in microbial loads between commercially available and home-prepared varieties [18, 20]. It is also established that during manure storage the detection and risk assessment of *L. monocytogenes* is complicated, as the organism is in a viable state but in an uncultivable form [21]. Hence, human might get exposed to this pathogen through the use of animal-based products like dairy, meat, etc., and also through handling and cultivation processes in the farm, thereby causing listeriosis infection. Listeriosis infection in humans is treated through the administration of antibiotics including penicillin, chloramphenicol, gentamicin, tetracycline, amoxicillin/ampicillin, rifamycin or trimethoprim and sulfamethoxazole as a mono or a combination therapy [22].

A growing concern for human health is the rapid rise in antibiotic resistance, largely driven by the overuse of antibiotics in livestock production and the treatment of various human diseases. There exists a significant knowledge gap regarding the role of animals and the consumption of animal-derived products as sources of drug-resistant pathogenic strains and their impact on human health. *Listeria* species are typically susceptible to a broad spectrum of antibiotics; however, the first multi-drug resistant (MRD) *L. monocytogenes* strain was identified in 1988 [23]. Since then, antibiotic-resistant (AMR) *L. monocytogenes* isolates have been reported frequently in food products, environmental sources, and human cases of listeriosis [24]. The presence of drug-resistant genes in *L. monocytogenes* and antibiotic resistance can significantly raise a major public health issue, mainly in the food industry. There are many reports on the emergence of resistant strains of *L. monocytogenes* isolated from dairy products [25].

*Listeria spp.* develops AMR that differs broadly among different strains, based on the origin and year of identification, utilization of antibiotics both in humans and livestock and regional variations. Currently in developing countries like India, there is limited data on the AST and prevalence of *Listeria* species [26]. Hence, surveillance of AMR in *L*. *monocytogenes* and other *Listeria* species is necessary. The precise, efficient and economical way of tracing the AMR organisms is through the use of multiple antibiotic resistance (MAR) index method. MAR index of individual bacterial isolates is generally calculated to determine the degree of contamination. If the MAR index value is above 0.2, it is indicated that there is an elevated level of contamination in an area that utilizes antibiotics routinely [22].

There is very little information available on the prevalence of *Listeria* spp. in the Himalayan region, where traditional foods like smoked meat, dried fish and fermented dairy products are consumed commonly. Sikkim, a biodiversity hotspot and an agro-tourism economy, depends on the locally produced and often minimally processed foods. The culinary practices of Sikkim’s diverse tribal communities involve a wide range of traditional food preparation and preservation methods. Despite minimal food exposure to contaminants and processing, there is a significant chance of the transmission and propagation of foodborne pathogens in hilly terrains due to different food practices.

Since the prevalence of *L. monocytogenes* contamination in the food supply chain of Sikkim is not thoroughly investigated, this study aims to address that gap by evaluating the prevalence, sources and AMR patterns of *L. monocytogenes* in various food samples. Our findings can help improve public awareness and bring preventive strategies into focus.

## Materials and methods

### Study area

**Fig. 1:**
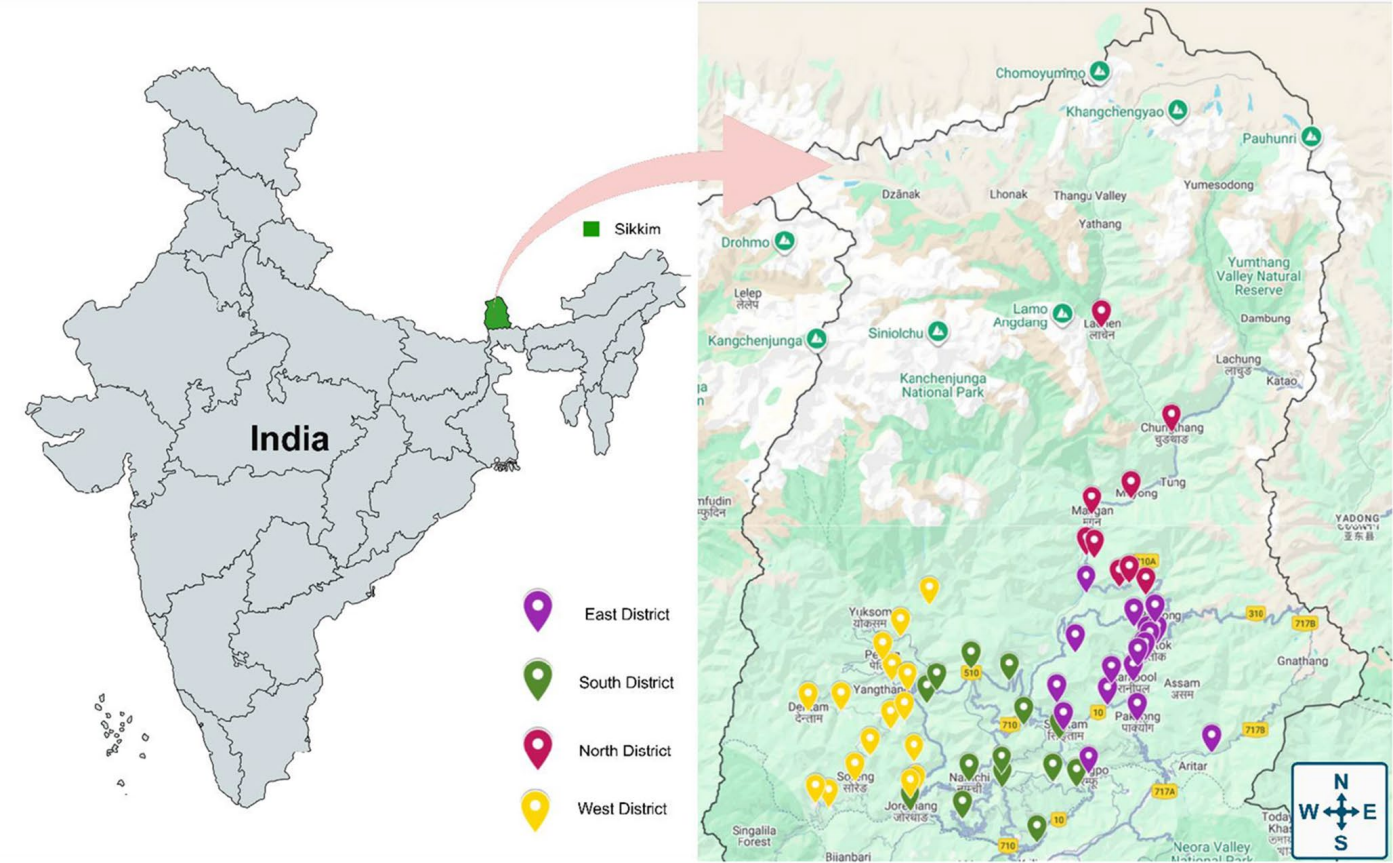
Map of Sikkim showing the study sampling area across four districts

The present investigation was carried out in the four districts of Sikkim (East, West, North and South), India. Sikkim is located at a topographical location of latitude 27.3516° North to 88.3239° East, as shown in Fig. 1. Regular field trips were conducted across various locations from April 2023 to May 2025. During this period, approximately 110 to 120 food samples were collected each month. These samples included raw/dry meat, cooked & un-cooked food items, milk and various milk products, street foods, fermented/preserved/local foods, beverages in the form of alcohol. Sampling locations and vendors were randomly selected within each site to ensure diversity, and care was taken to avoid collecting repeated samples from the same point. In total, the study covered 726 unique sampling points.

### Sample size

A total of 2829 samples of various foods from local shops, hotels, restaurants, butchers, abattoirs and local households selling local ethnic foods were collected at different locations and towns within Sikkim. After collection, food samples were transported to the laboratory in an ice pack box and stored at 4 °C to ensure same-day testing.

### Sample collection

Food samples (20–50 g solid/20 mL liquid) were collected aseptically in sterile containers (plastic zip-lock bags or sterile jars). Solid samples were homogenized in a sterile mortar, while liquid samples were directly diluted in Ringer’s lactate solution and incubated for 24 h at 37 °C. Samples were tested for the presence of *L. monocytogenes* using the conventional bacterial culture and biochemical identification methods following the standard methods [27].

### Enrichment and isolation protocol

A two-stage enrichment method was used to isolate *L. monocytogenes*. For primary enrichment, 1 mL of homogenized sample was inoculated into 9 mL of half-strength Fraser broth (HiMedia) with Fraser Selective Supplement (HiMedia, Mumbai) and incubated at 30 °C for 24 h. Secondary enrichment involved transferring 0.1 mL from the primary culture into 10 mL of full-strength Fraser broth (supplemented) and incubating at 37 °C for 48 h. Enriched samples were then streaked onto polymyxin acriflavin lithium-chloride ceftazidime esculin mannitol (PALCAM) agar (HiMedia) with selective Supplement (HiMedia) and incubated at 37 °C for 48 h. Colonies exhibiting characteristic gray-green colour with a diffused black zone were selected for further biochemical confirmation.

### Biochemical confirmation

Presumptive *L. monocytogenes* isolates were confirmed through a series of biochemical tests: Colonies showing catalase activity were assessed using 3% H₂O₂ (catalase-positive), and while oxidase testing yielded negative results, they were inoculated in Brain-Heart Infusion broth (HiMedia) at 25 °C for 12–18 h. For further biochemical tests, isolates were streaked on Triple Sugar Iron (TSI) agar to test carbohydrate fermentation patterns, with urease activity evaluated in Christensen’s urea medium. Additional tests included Simmons citrate utilization, nitrate reduction, indole production (Kovacs reagent), Methyl Red-Voges Proskauer (MR-VP) reactions, and motility (hanging drop), medium. Lysine and arginine metabolism were examined using Lysine Iron Agar (LIA). All tests were interpreted after 48 h incubation at 37 °C [27].

### VITEK 2 system

Phenotypically identified *L. monocytogenes* isolated that were confirmed biochemically were further subjected to VITEK 2 Compact (bioMérieux, France) with the GP ID card for further confirmation. Results were interpreted according to the manufacturer’s guidelines.

### Multiplex PCR Assay for confirmation of *L. monocytogenes*

The p60 antigen is a hydrolase protein encoded in the gene *iap* that is present in all the *L. monocytogenes* serotypes. Similarly, the zinc-dependent metalloprotease (encoded by the gene *mpl*) is involved in the virulence of this pathogen. In this study, these two genes were targeted to confirm *L. monocytogenes* isolated from different sources. The PCR assay was first performed using genomic DNA extracted from reference *L. monocytogenes* strain, which has been used as a positive control. DNase-free distilled water was used as a negative control.

Biochemically identified *L. monocytogenes* isolates were inoculated into LB agar (HiMedia) and incubated overnight at 37 °C. Bacteria lysate was prepared by resuspending one colony in 30 𝜇L of deionized water, boiled for 5 min, followed by centrifuging at 8000 ×g for 2 min. About 2 𝜇L of supernatant was used as the DNA template in the PCR assay.

Multiplex PCR was performed using the published protocol [28, 29] with modifications. The components used in the PCR include 10 pmol/µl of each primer; 1 × (12.5 µl) ready to use GoTaq Green master mix (Promega, USA) and the final reaction volume was adjusted to 25 µl. The PCR was performed using Mastercycler Nexus Gradient (Eppendorf, Germany). Prime sequences, PCR conditions, and the expected amplicon sizes are shown in (supplementary table S1). The PCR products were analyzed by electrophoresis on 1.5% agarose gels with 10 mg/mL ethidium bromide (Sigma, USA) that were run at 100 V for 60 min. PCR products were visualized under a UV transilluminator and photographed using an image analyzer (BioRad, Hercules, CA, USA).

### Antimicrobial susceptibility test (AST)

AST was performed on all *L. monocytogenes* isolates identified from food samples using the microbroth dilution technique to determine the minimum inhibitory concentrations (MICs), following guidelines established by the Clinical and Laboratory Standards Institute [30, 31]. The procedure followed the standards outlined in both the CLSI M100 and M45 documents. A panel of 11 antibiotics was tested, including ampicillin (AMP), penicillin (PEN), trimethoprim-sulfamethoxazole (SXT), and meropenem (MRP), which are covered under the CLSI M45 guidelines for *Listeria* spp. Since specific interpretive criteria for chloramphenicol (CHL), ciprofloxacin (CIP), gentamicin (GEN), tetracycline (TET), erythromycin (ERY), clindamycin (CLI), and vancomycin (VAN) are not provided for *L. monocytogenes* in CLSI documents, susceptibility results for these agents were interpreted using the breakpoints established for *Staphylococcus* spp., as described in a previous study [32].

### Multiple antibiotic resistance (MAR) index

The MAR index of *L. monocytogenes* isolates was calculated following the method described by Manyi-Loh et al. [22]. The MAR index was determined using the formula MAR = a/b, where (a) represents the number of antibiotics to which an isolate was resistant, and (b) is the total number of antibiotics tested in this study (*n* = 11). Based on this approach, an MAR index value ≥ 0.2 suggests that the isolate originated from an environment where antibiotics are frequently used or uncontrolled antibiotic use and is considered MAR, while a value ≤ 0.2 indicates limited exposure to antimicrobials.

###  Quality control and ethical considerations

Reference strains (*L. monocytogenes* ATCC 31152) were included in each batch, provided by ICMR-NIRBI Kolkata, the External Quality Control (EQC) Center. The study was approved by the Ethics Committee of Sikkim Manipal University, Gangtok, India (approval number: SMIMS/IEC/2019 − 116) under the ICMR task force study. Sample collection permit was obtained from the Health & Family Welfare Department, Government of Sikkim (No: 847/FSS Cell/H&FW/2020).

## Results

### Overall Prevalence of *L. monocytogenes*

A total of 2829 food samples were screened for the presence of *L. monocytogenes* during the study period. Of these, 98 samples tested positive, resulting in an overall prevalence rate of **3.5%.** The distribution of positive samples varied across different food categories, including raw/dried meat, cooked items, uncooked items, street foods, fermented/preserved items, milk and milk products and beverages.

### Identification of *L. monocytogenes* in food categories

The highest proportion of *L. monocytogenes* isolates was recovered from cooked food items, which accounted for 39 (5.1%) of the 758 tested samples. Within this category, the most frequently contaminated item was cooked vegetables (*n* = 16), followed by chicken (*n* = 8), rice (*n* = 6), and smaller counts in beef (*n* = 2), pork (*n* = 2), dal (*n* = 2), eggs (*n* = 1), fish (*n* = 1), and mutton (*n* = 1). Raw or dried meat samples (*n* = 481) yielded 23 positives (4.8%), with contamination highest in chicken (*n* = 10) and fish (*n* = 8), followed by beef (*n* = 2), buffalo meat (*n* = 2), and pork (*n* = 1). These results underscore the exposure of raw animal products to microbial contamination, especially in regions with limited cold chain infrastructure. Fermented and preserved foods represented 15 positive cases out of 499 samples (3.0%). Contaminated items included gundruk (dry mustard leaves) (*n* = 2), kinema (fermented soya bean) (*n* = 2), sinki (dry mustard shoot and radish) (*n* = 2), khuri (buckwheat rolls) (*n* = 2), kentsong (roasted maize) (*n* = 2), and one positive sample each from zhero (deep fried rice flour), wachipa (burnt feather of hen cooked with rice, chicken, meat, and vegetables), nakima (tupistra nutans veritable/pickles), tama (bamboo shoots) and millet bread. These traditionally prepared foods are often produced without standardized microbial safety controls, which may contribute to contamination. Milk and milk products accounted for 7 positive cases (3.02%) out of 232 samples tested. The contaminated items were sweets (*n* = 4), boiled milk (*n* = 2), and churpi (*n* = 1), reflecting a moderate but consistent risk in both fresh and processed dairy products. Street food samples (*n* = 298) showed a positivity rate of 2.68% (8 samples), with momo (*n* = 5) and noodles (*n* = 3) being the identified sources. Uncooked food items, such as dough and vegetables, had the lowest detection rate at 0.92% (5 out of 545 samples), including maida dough (*n* = 4) and raw vegetables (*n* = 1). Despite the low prevalence, the presence of *L. monocytogenes* in uncooked products highlights potential risks from improper handling or environmental contamination. Finally, among beverages, only 1 sample tested positive (6.25%) out of 16, particularly from a locally fermented alcoholic drink, chaang. Though this category had the highest percentage, the sample size was limited as shown in Table 1.

**Table 1:** The prevalence rates by food category

Food Category	Samples Tested	Positive (%)
Raw/dried meat	481	23 (4.8%)
Cooked items	758	39 (5.1%)
Uncooked items	545	05 (0.9%)
Street food	298	08 (2.7%)
Fermented/preserved food	499	15 (3.0%)
Milk and milk products	232	07 (3.0%)
Beverages	16	01 (6.2%)

### District-wise trends

The distribution of *L. monocytogenes* positive samples across the four districts of Sikkim revealed notable regional variations and food-specific contamination patterns. South Sikkim reported the highest number of positive cases (31 out of 98; 31.63%), with contamination detected in raw meats, cooked vegetables and multiple fermented items, indicating diverse sources of exposure. North Sikkim accounted for 24 cases (24.49%), with notable contamination in fermented foods such as kinema, sinki, and kentsong, as well as in cooked vegetables and meats, suggesting risks associated with traditional preparation methods. East Sikkim recorded 23 positive cases (23.47%), primarily in cooked vegetables, chicken, and dal, along with some raw and fermented foods, reflecting a broad range of contamination points. West Sikkim had the lowest number of positives (*n* = 20; 20.41%) but still showed contamination in cooked rice, vegetables, fermented products, and street foods as mentioned in Table 2.

**Table 2:** Distribution of *Listeria monocytogenes*-Positive food samples by category and district in Sikkim

Items	Category	East (23)	West (20)	North (24)	South (31)	Total
Raw/dried meat	Chicken	4	1	2	3	10
	Fish	-	2	3	3	8
	Beef	-	-	-	2	2
	Buff	2	-	-	-	2
	Pork	-	-	1	-	1
Cooked items	Vegetables	4	3	6	3	16
	Chicken	-	3	4	1	8
	Rice	1	4	1	-	6
	Beef	-	1	1	-	2
	Eggs	1	-	-	-	1
	Dal	2	-	-	-	2
	Pork	1	-	-	1	2
	Fish	1	-	-	-	1
	Mutton	-	-	-	1	1
Uncooked items	Dough	2	1	-	1	4
	Vegetables	-	-	-	1	1
Street food	Momo	1	1	1	2	5
	Noodles	-	1	1	1	3
Fermented/preserved food	Gundruk	1	1	-	-	2
	Kinema	-	-	2	-	2
	Zhero	-	1	-	-	1
	Wachipa	1	-	-	-	1
	Tama	-	-	1		1
	Sinki	-	-	-	2	2
	Nakima	1	-	-	-	1
	Millet bread	-	-	-	1	1
	Khuri	-	-	-	2	2
	Kentsong	-	-	-	2	2
Milk and milk products	Sweets	1	1	-	2	4
	Boiled milk	-	-	1	1	2
	Churpi	-	-	-	1	1
Beverages	Alcohol Chaang	-	-	-	1	1

### Seasonal distribution

**Fig. 2:**
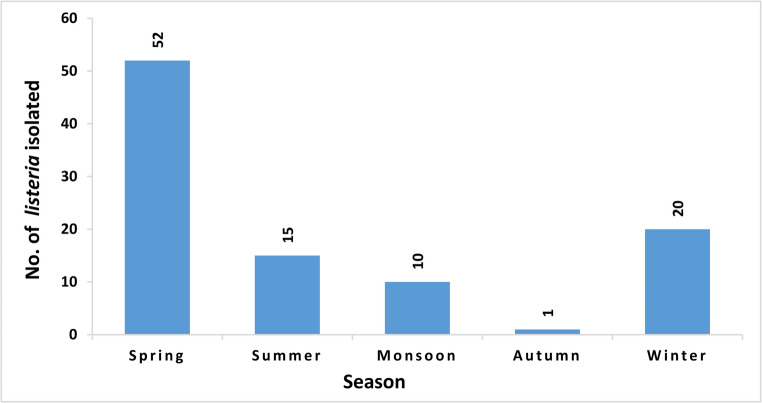
Seasonal wise contamination of *Listeria monocytogenes*

The occurrence of *L. monocytogenes* showed significant seasonal variation over the sampling period. The highest number of isolates was detected during the spring season (*n* = 52), accounting for the majority of total isolates. This was followed by winter season with (*n* = 20) isolates, summer season with (*n* = 15), monsoon season (*n* = 10) and the autumn season recording the lowest number of isolates (*n* = 1) as shown in Fig. 2.

### Multiplex PCR

All biochemically confirmed *L. monocytogenes* isolates (*n* = 98) were further validated using a multiplex PCR assay targeting the *iap* and *mpl* genes. The amplification produced distinct bands of 454 bp (*iap*) and 679 bp (*mpl*) in all tested isolates, consistent with the expected product sizes and the positive control strain (*L. monocytogenes* ATCC 31152). No amplification was observed in the negative control, confirming assay specificity and reliability. These results conclusively verified the identity of all isolates as *L. monocytogenes*.

### Antimicrobial susceptibility

*L. monocytogenes* isolated from food samples were subjected to AST using the microbroth dilution method. Among the β-lactam antibiotics, penicillin and ampicillin showed susceptibility in 60% and 58% of isolates, respectively, with resistance observed in 38% and 42% (Table 3). For quinolones, ciprofloxacin demonstrated high efficacy, with 93% of isolates susceptible and only 2% resistant. Gentamicin, an aminoglycoside, showed a susceptibility rate of 87% and lower resistance of 6%. Among the amphenicol, chloramphenicol exhibited 80% susceptibility. Erythromycin showed moderate activity, with 55% susceptibility and 45% resistance. Notably, clindamycin had the lowest susceptibility rate, with only 16% of isolates classified as susceptible, and nearly half (48%) exhibiting resistance. For meropenem 82% of isolates were susceptible, with 18% resistant. Tetracycline and trimethoprim-sulfamethoxazole showed excellent activity, each with 98% susceptibility and minimal or no resistance. Vancomycin showed moderate efficacy, with 60% susceptibility and a notable resistance rate of 37%.

**Table 3:** Antimicrobial susceptibility test results of *Listeria monocytogenes*

Antibiotic Class	Antibiotic	MIC (µg/mL) interpretive Criteria			No. of isolates (%)			CLSI Guideline Used
		Susceptible	Intermediate	Resistant	Susceptible	Intermediate	Resistant	
Penicillin	Penicillin	≤ 2	-	-	59 (60%)	2 (2%)	37 (38%)	M45 (*Listeria* spp.)
	Ampicillin	≤ 2	-	-	57 (58%)	-	41 (42%)	M45 (*Listeria* spp.)
Quinolones	Ciprofloxacin	≤ 1	2	≥ 4	91 (93%)	5 (5%)	2 (2%)	M100 (*Staphylococcus* spp.)
Aminoglycosides	Gentamicin	≤ 4	8	≥ 16	85 (87%)	7 (7%)	6 (6%)	M100 (*Staphylococcus* spp.)
Amphenicols	Chloramphenicol	≤ 8	16	≥ 32	78 (80%)	12 (12%)	8 (8%)	M100 (*Staphylococcus* spp.)
Macrolides	Erythromycin	≤ 0.5	1–4	≥ 8	54 (55%)	-	44 (45%)	M100 (*Staphylococcus* spp.)
Lincomycin	Clindamycin	≤ 0.5	1–2	≥ 4	16 (16%)	34 (35%)	47 (48%)	M100 (*Staphylococcus* spp.)
Carbapenems	Meropenem	≤ 0.25	-	-	80 (82%)	-	18 (18%)	M45 (*Listeria* spp.)
Tetracyclines	Tetracycline	≤ 4	8	≥ 16	96 (98%)	2 (2%)	-	M100 (*Staphylococcus* spp.)
Glycopeptides	Vancomycin	≤ 2	4–8	≥ 16	59 (60%)	3 (3%)	36 (37%)	M100 (*Staphylococcus* spp.)
Combinations	Trimethoprim-Sulfamethoxazole	≤ 0.5/9.5	-	-	96 (98%)	-	2 (2%)	M45 (*Listeria* spp.)

**Fig. 3:**
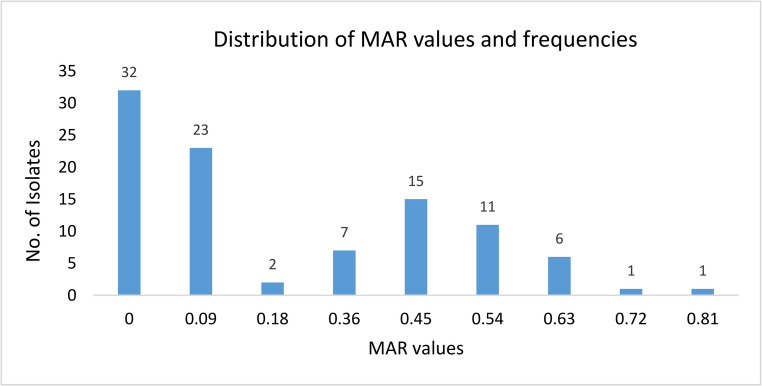
Distribution of MAR Values and frequencies of *Listeria monocytogenes*

In our study, the MAR values range from 0 to 0.81. We know that anything > 0.2 indicates MAR and employs bacterial isolates exhibiting resistance to three or more classes of antibiotics. Here we observe that the number of *L. monocytogenes* isolates with > 0.2 MAR values was 41 out of 98, which presents a prevalence rate of 41.8% as observed in Fig. 3. Several isolates showed resistance to four or more antibiotics, demonstrating clear multidrug-resistant (MDR) characteristics as showed in Table 4.

**Table 4:** Listeria isolates resistant to four or more antibiotics

Number of antibiotics	Number of Resistant Isolates	MAR Phenotypes
9	1	AMP, E,V, G,P, CIP, C,M, COT
8	1	AMP, E,V, G,P, CIP, C,M
7	5	AMP, E,V, P,C, M,CD
7	1	AMP, E,V, G,P, CD, M
6	5	AMP, E,V, P,CD, M
6	3	AMP, E,V, G,P, CD
6	1	AMP, E,V, P,COT, CD
6	1	AMP, E,V, P,C, M
5	8	AMP, E,V, P,CD
5	8	AMP, E,V, P,M
5	1	AMP, E, V, M, CD
4	3	AMP, E, P, M
4	2	AMP, E, V, P
4	1	AMP, E, M, CD

*AMP* ampicillin, *CIP* ciprofloxacin, *C* chloramphenicol, *COT* Co-trimoxazole, *CD* Clindamycin, *E *erythromycin, *Ggentamicin, P* penicillin, *M* meropenem, *V* Vancomycin

## Discussion

*L. monocytogenes* is a food-borne pathogen, ubiquitous in nature and is most often found in different environments including soil, water, livestock industry (like animal fodder, meat, and dairy products), sewage and faeces of humans and animals [22]. This study presents the first region-wide assessment of *L. monocytogenes* contamination across various food categories in the Himalayan state of Sikkim, India. Presence of *L. monocytogenes* in different foods underscore its public health importance. The pathogen was detected across a wide range of food types, with the highest proportion mainly in cooked foods, followed by raw/dried meat, fermented/preserved foods. Regular monthly sampling from 726 unique points across various seasons showed a higher incidence observed in certain months that may influence bacterial survival and growth. The unexpectedly high detection of *L. monocytogenes* in cooked foods and street foods suggests a strong likelihood of post-processing contamination, possibly during cooling, storage, or handling. Similar observations have been made in studies conducted in other parts of India and globally, where improperly stored ready-to-eat (RTE) foods were identified as key vectors for survival and proliferation of *Listeria* spp [16, 33, 34]. This finding confirms the need for better hygiene standards and temperature control during storage and sale/delivery. The presence of the pathogen in raw and dry meats (especially chicken and fish), as well as in fermented products like kinema, gundruk, khuri, kentsong and sinki aligns with prior research indicating that animal-derived and traditionally fermented foods are high-risk categories [35]. Traditional fermentation often lacks standard microbial testing and sanitary regulation, especially about the presence of *L. monocytogenes*.

Milk and milk products like sweets, ready-to-consume milk, and churpi, reflect a moderate but consistent risk in both fresh and processed dairy products. *Listeria* spp. in street food like momo and noodles indicates a need for improved hygiene practices among food vendors. These observations are supported by studies conducted in North Karnataka and elsewhere, where *L. monocytogenes* was also found in dairy, fermented, and street foods [17, 25]. Spatial analysis revealed important regional disparities. South Sikkim recorded a higher number of positive cases, with higher contamination of raw/dried meat and fermented items. North Sikkim, despite being less urbanized, showed a notable incidence of *Listeria* spp. in cooked items, raw/dried meat and fermented foods. This suggests the role of household-level hygiene practices leading to food contamination. East Sikkim followed closely, with a high occurrence of *L. monocytogenes* in cooked items and raw/dried meats. West Sikkim had the lowest prevalence, although isolated cases in raw fish and fermented vegetables indicate that risk exists across the state. These findings emphasize the importance of district-specific surveillance and localized food safety strategies, especially in areas where traditional foods and preparation methods are culturally followed and widely consumed. The state’s humid subtropical climate and variable storage practices may also increase the risk of microbial contamination [36]. The seasonal trend observed in the current study reveals a marked variation in the prevalence of *L. monocytogenes* across different seasons in Sikkim, with the highest number of isolates recorded during spring, followed by winter. The peak in spring may be due to favourable environmental conditions, moderate temperature and humidity which support the survival and growth of *Listeria* spp.in food products [37, 38].

The AMR patterns observed in *Listeria* spp. isolates highlight growing concerns in foodborne pathogen management. In our study, moderate to high resistance was noted against first-line antibiotics like ampicillin and penicillin, which are typically used in listeriosis therapy. This aligns with other findings [39]. Resistance to clindamycin and erythromycin was also notably high in our study, consistent with a previous report [40]. Ciprofloxacin and gentamicin seem effective drugs. Resistance of some of the isolates to these drugs underscores the importance of continuous AMR monitoring. The observed resistance to vancomycin, although not a first-line drug for *Listeria*, is noteworthy from a public health perspective and has been previously linked to gene transfer and environmental exposure [41]. Conversely, high susceptibility to tetracycline and trimethoprim-sulfamethoxazole is reassuring, reflecting similar efficacy reported in other studies [24]. In our study, *L. monocytogenes* showed remarkable (98%) susceptibility to tetracycline. Overall, our findings highlight the importance of emerging MDR *L. monocytogenes* in different food sources and underscore the need for rational antibiotic use and inclusion of this pathogen in routine food microbial quality testing.

The confirmation of *L. monocytogenes* isolates using multiplex PCR targeting the *iap* and *mpl* genes provided additional molecular validation to the phenotypic and biochemical identification. Both *iap* and *mpl* are conserved virulence-associated genes, essential for invasion and intracellular survival, and are consistently reported among *L. monocytogenes* isolates from various food sources [42]. The *iap* gene encodes the invasion-associated p60 antigen, a key marker for species-level identification, while *mpl*, a zinc-dependent metalloprotease, plays a role in activating phospholipases and pathogenesis. The detection of these two virulence genes across all biochemically identified isolates in this study corroborates the molecular identity of the pathogen and reflects its potential virulence, consistent with reports from other global and Indian studies on foodborne *L. monocytogenes*. These findings highlight that *L. monocytogenes* strains contaminating foods in Sikkim possess classical virulence determinants, emphasizing the necessity of continued monitoring to prevent possible public health risks associated with contaminated ready-to-eat and raw foods.

The routine foodborne pathogen surveillance and monitoring of AMR not only provide the extent of the resistance spectrum; but also contribute to assessing the validation of several control measures to overcome the emerging crisis. The utilization of antibiotics for bulk production of animal-based products like meat, eggs, dairy products etc., at a sub-therapeutic level has encouraged the evolution and sustenance of MAR organisms found in livestock. The MAR index values more than 0.20, strongly signifies that these organisms had frequent antibiotic exposure [22].

The calculated MAR index values (0.09–0.81) and the proportion of isolates exceeding 0.2 indicate the possible overuse or misuse of antibiotics in local agricultural and food-handling environments. Comparable MAR values have been reported in *Listeria* isolates from vegetables, dairy, and meat products [22]. The co-resistance patterns observed commonly involving ampicillin, erythromycin, vancomycin, and penicillin suggest the presence of multidrug-resistant phenotypes that may compromise treatment efficacy.

The occurrence of MAR values above 0.2 implies that *L. monocytogenes* may have originated from environments contaminated with antibiotic residues or waste from livestock farming, aquaculture, or hospital effluents. Horizontal gene transfer from other Gram-positive bacteria such as *Enterococcus*, *Streptococcus*, and *Staphylococcus* could also contribute to the acquisition of resistance determinants [23, 41] These findings emphasize the urgent need for antibiotic stewardship in food production systems, regular surveillance of resistance profiles, and enforcement of hygienic foodhandling practices to prevent dissemination of MDR *Listeria* strains in the food chain.

The detection of the *L. monocytogenes*, though low in scale, the commonly consumed cooked items often cause a higher risk of infection. Additionally, the varied presence across diverse foods and districts underscores the need for improved food safety training for vendors and handlers, better enforcement of hygiene regulations, and region-specific consumer awareness campaigns. This study not only fills a critical data gap for Sikkim, but also offers a framework for other Northeastern states of India with similar dietary habits and infrastructural challenges. Future work should focus on molecular typing of isolates to trace contamination pathways and screen the genes responsible for virulence, biofilm formation and antibiotic resistance, as these factors play pivotal roles in infection, long-term survival and treatment challenges.

### Limitations

This study provides a comprehensive overview of *L. monocytogenes* prevalence and AMR in various food commodities across Sikkim. Although molecular confirmation through multiplex PCR targeting the *iap* and *mpl* genes strengthened isolate identification, advanced genomic characterization such as whole-genome sequencing (WGS) or multilocus sequence typing (MLST) was not performed. These approaches could further clarify strain diversity and virulence profiles. Future studies incorporating molecular tools and environmental assessments are recommended to build a more detailed epidemiological profile of *L. monocytogenes* in the region.

## Conclusion

This study provides valuable insight into the presence of *L. monocytogenes* in a wide range of commonly consumed foods across Sikkim. Although the overall prevalence was modest, the detection of this pathogen in cooked foods, raw/dried meats, and fermented/preserved foods gives a clear message, i.e., food safety cannot be assumed safe, even in seemingly familiar products. The presence of *L. monocytogenes* in cooked vegetables and milk products is concerning, as these are often consumed by vulnerable groups such as children and the elderly. The findings highlight how traditional food practices, if not handled with proper hygiene, can become potential routes of contamination. Likewise, the seasonal and district-wise variations point to the importance of environmental and regional factors that influence microbial risks. These insights call for focused food safety education, stronger monitoring systems, and community level awareness especially during the spring season and in high-risk areas like South and North Sikkim. Food is deeply tied to culture and livelihood in the Himalayan region. Our aim is to highlight the need to strengthen traditional food practices with safe handling techniques. With small, consistent efforts from households to health departments, it is possible to ensure the food supply chain and safeguard the health of our communities. This study serves as a starting point toward this goal.

## Supplementary Information

Below is the link to the electronic supplementary material.

Supplementary Material 1.

Supplementary Material 2.
