# Supplementary material for: Exploring the prevalence and antibiotic resistance of Listeria monocytogenes in diverse food commodities across Sikkim, India
Source: Braz J Microbiol. 2025 Dec 23;57(1):23. doi: 10.1007/s42770-025-01853-0 (PMC12728136; doi:10.1007/s42770-025-01853-0)
Supplement: Supplementary file 2 — Supplementary Material 2. [file 42770_2025_1853_MOESM2_ESM.docx]

**Table S1** PCR primers and conditions for the identification of *L. monocytogenes*

| **Primer (5’-3’)** | **Target gene (amplicon bp)** | **PCR Condition** | **Reference** |
| --- | --- | --- | --- |
| LMp-F: CTGGCACAAAATTACTTACAACGA  LMp-R: AACTACTGGAGCTGCTTGTTTTTC | *iap* variant (454) | Initial Denaturation at 94°C for 5 min  ***35 cycles of***  Denaturation at 94 °C for 1 min  Annealing at 60°C for 1 min  Extension at 72°C for 1 min  Final extension 72°C for 10 min | 28 |
| LM20-F: TGATGAAATAAAGGTCCACG  LM20-R: CAAGCCATAATGAACAAACG | *mpl (*679) |  | 29 |
